# Vision-Based Traffic Data Collection Sensor for Automotive Applications

**DOI:** 10.3390/s100100860

**Published:** 2010-01-22

**Authors:** David F. Llorca, Sergio Sánchez, Manuel Ocaña, Miguel. A. Sotelo

**Affiliations:** Electronics Department, University of Alcalá, Polytechnic School, University Campus, Alcalá de Henares, Madrid 28871, Spain; E-Mails: sergio.sanchez@depeca.uah.es (S.S.); mocana@depeca.uah.es (M.O.); sotelo@depeca.uah.es (M.A.S.)

**Keywords:** automotive sensor, vehicle detection, computer vision, distance accuracy

## Abstract

This paper presents a complete vision sensor onboard a moving vehicle which collects the traffic data in its local area in daytime conditions. The sensor comprises a rear looking and a forward looking camera. Thus, a representative description of the traffic conditions in the local area of the host vehicle can be computed. The proposed sensor detects the number of vehicles (*traffic load*), their relative positions and their relative velocities in a four-stage process: *lane detection*, *candidates selection*, *vehicles classification* and *tracking*. Absolute velocities (*average road speed*) and global positioning are obtained after combining the outputs provided by the vision sensor with the data supplied by the CAN Bus and a GPS sensor. The presented experiments are promising in terms of detection performance and accuracy in order to be validated for applications in the context of the automotive industry.

## 1. Introduction

Developing onboard vehicle detection sensors aimed at improving the safety of road users is one of most important topics in the context of automotive applications and it has attracted a lot of attention during the last decade. Vehicle detection is a very challenging task due to the high intra-class variability of vehicle appearance. Vehicles may vary in shape, color and size, and their appearance is highly affected by pose, nearby objects and illumination conditions. Vehicle detection systems have many applications in the context of the automotive industry such as platooning, Adaptive Cruise Control (ACC), forward/rear collision avoidance and mitigation, traffic detection, Floating Car Data (FCD), *etc*. Robust and accurate vehicle detection is a crucial step in all these systems.

The most common approach to vehicle detection has been carried out by using active sensors such as acoustic-based [1], radar-based [2] and laser-based [3,4]. However, passive sensors, and more specifically optical sensors, have attracted most of the attention of the research community as well as the industry, due to two main aspects: inexpensive cost and new potential applications (Lane Departure Warning (LDW), pedestrian detection, traffic sign recognition, *etc.*). We refer to [5] for general background concerning vehicle detection, covering both active and passive sensors.

In this paper we present a daytime traffic data collection sensor for automotive applications which comprises both forward and rear facing inexpensive cameras operating in the visible spectrum. Compared to previous extended FCD systems [6,7] the proposed approach provides a more representative description of the local traffic conditions of the host vehicle, since it covers a nearly 360 degree field of view. The proposed sensor detects the number of vehicles (*traffic load*), their relative positions and their relative velocities in a four-stage process: *lane detection*, *candidates selection*, *vehicles classification* and *tracking*. Then, absolute velocities (*average road speed*) and global positioning are obtained after combining the outputs provided by the vision sensor with the data supplied by the CAN Bus and a GPS sensor. The sensor is mainly designed to supply data corresponding to both road traffic load and speed. However, the accuracy of the host-to-vehicle distances estimated by the proposed approach enables its use for other automotive applications (ACC, collision avoidance, *etc.*) without the need of other sensors [8].

The rest of the paper is organized as follows; Section 2 provides the description of the vision-based traffic detection sensor including the analysis of the error in the estimation of the host-to-vehicle distance. Section 3 is dedicated to experimental results and finally Section 4 summarizes the conclusions and future work.

## 2. Vision-Based Traffic Detection Sensor

### 2.1. Architecture description

**Figure 1.**
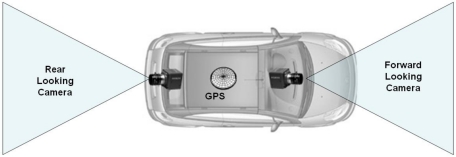
Traffic data collection sensor comprising two cameras and a GPS.

The proposed traffic collection sensor comprises two FireWire cameras: one rear looking camera and another forward looking one. Thus, the sensor range covers the local environment of the host vehicle enabling a nearly 360 degree field of view with the exception of the side blind areas (see Figure 1). A common hardware trigger synchronizes the image acquisition of both cameras and an onboard PC houses the computer vision software.

**Figure 2.**
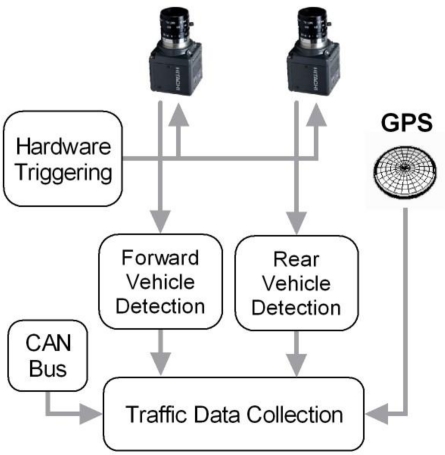
Overview of the traffic data collection sensor architecture.

Each individual vehicle detection system provides information about the number of detected vehicles and both their relative position and speed. These results are combined with the GPS measurements and the data provided by the CAN bus (vehicle speed) in order to provide globally referenced traffic information. Note that for vehicles without CAN bus interface, the vehicle speed can be computed from GPS measurements. This scheme is described in Figure 2.

The layers of the proposed architecture of both vision modules are conceptually the same: *lane detection*, *vehicle*-*candidates selection*, *vehicle recognition* and *tracking*. The first step of each one of the vision systems consists of reducing the searching space in the image plane in an intelligent manner in order to increase the performance of the vehicle detection module. Accordingly, road lane markings are detected and used as the guidelines that drive the vehicle searching process. The area contained by the limits of the lanes is scanned in order to find vehicle candidates that are passed on to the vehicle recognition modules. Thus, the rate of false positives is reduced. In case that no lane markings are detected, a basic *region of interest* is used instead covering the front, rear and side parts of the vehicle. Finally, a tracking stage is implemented using Kalman filtering techniques.

### 2.2. Lane detection

An attention mechanism is necessary in order to filter out inappropriate candidate windows based on the lack of distinctive features, such as horizontal edges and vertical symmetrical structures, which are essential characteristics of road vehicles. This has the positive effect of decreasing both the total computation time and the rate of false positive detections. Lane markings are detected using gradient information in combination with a local thresholding method which is adapted to the width of the projected lane markings. Then, clothoid curves are fitted to the detected markings. The algorithm scans up to 25 lines in the candidates searching area, from 2 meters in front of the camera position to the maximum range in order to find the lane marking measurements. The proposed method implements a non-uniform spacing search that reduces certain instabilities in the fitted curve. The final state vector is composed of six variables [9] for each lane on the road:

(1) $$x={\left[c_{0h},\;c_{1h},\;c_{\mathrm{ov}},\;c_{1v},\;x_{0},\;\theta_{0},\;w_{0}\right]}^{T}$$

where *c_0h_* and *c_1h_* represent the clothoid horizontal curvature parameters, *c*_0*v*_ and *c*_1*v*_ stand for the clothoid vertical curvature parameters, while *x*_0_, *θ*_0_ and *w*_0_ are the lateral error and orientation error with regard to the centre of the lane and the width of the lane respectively. The clothoid curves are then estimated based on lane marking measurements using a Kalman filter [10] for each lane.

**Figure 3.**
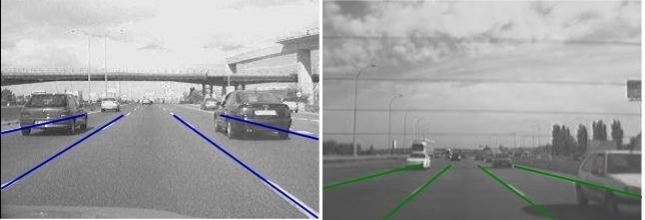
Vehicle searching area as a result of the lane markings analysis for forward and rear modules.

Apart from the detected road lanes additional virtual lanes have been considered so as to cope with situations in which a vehicle is located between two lanes (for example, if it is performing a lane change manoeuvre). Virtual lanes provide the necessary overlap between lanes, avoiding both misdetections and double detections caused by the two halves of a vehicle being separately detected as two potential vehicles. A virtual lane is located to provide overlap between two adjoining lanes. Figure 3 provides some examples of lane markings detection in real outdoor scenarios. Detected lanes determine the vehicle searching area and help reduce false positive detections. In case no lane markings are detected by the system, fixed lanes are assumed instead.

### 2.3. Forward and rear vehicle detection

**Figure 4.**
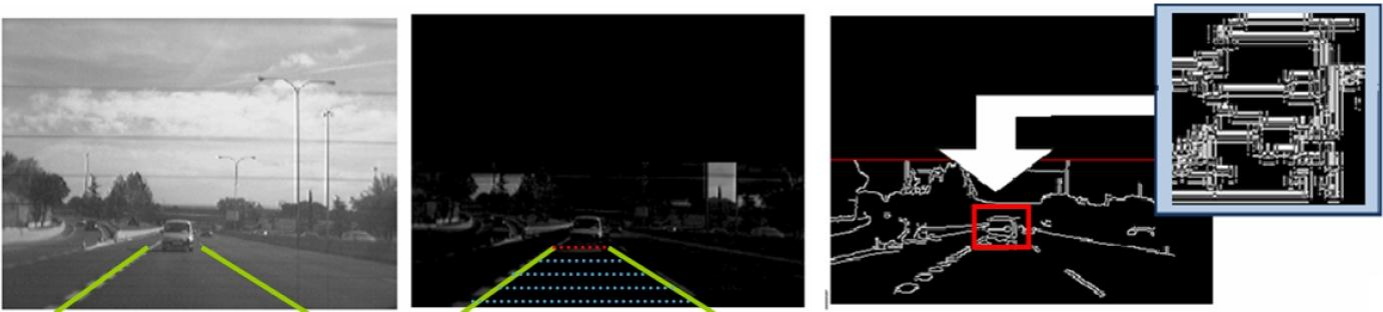
From left to right: original image; contact point detection on white top-hat image; candidate pre-selected with high entropy of Canny points.

Forward and rear looking vehicle detection systems share the same algorithmic core. The attention mechanism sequentially scans each road lane from the bottom to the maximum range looking for a set of features that might represent a potential vehicle. Firstly, the vehicle contact point is searched by means of the top-hat transformation. This operator allows the detection of contrasted objects on non-uniform backgrounds [11]. There are two different types of top-hat transformations: white hat and black hat. The white hat transformation is defined as the residue between the original image and its opening. The black hat transformation is defined as the residue between the closing and the original image. The white and black hat transformations are analytically defined as follows:

(2) $${\mathrm{WH}}_{T}(x,\;y)=\left(f-f\circ b\right)(x,\;y)\;\text{White Hat}$$

(3) $${\mathrm{BH}}_{T}(x,\;y)=\left(f•b-f\right)(x,\;y)\;\text{Black Hat}$$

where ○ denotes the opening operator and • means for the closing operator. In our case we use the white hat operator [Equation (2)] since it enhances the boundary between the vehicles and the road [12]. Horizontal contact points are pre-selected if the number of white top-hat features is greater than a configurable threshold. Then, candidates are pre-selected if the entropy of Canny points is high enough for a region defined by means of perspective constraints and prior knowledge of target objects (see Figure 4).

**Figure 5.**
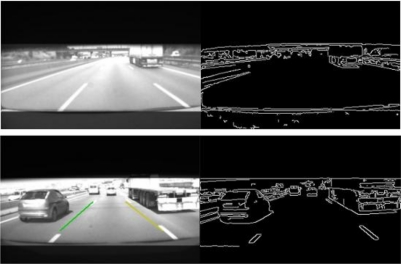
Canny images after adaptive thresholding.

Before computing the Canny features, an adaptive thresholding method is applied. This process is based on an iterative algorithm that gradually increases the contrast of the image, and compares the number of Canny points obtained in the contrast increased image with the number of edges obtained in the current image. If the number of Canny features in the actual image is higher than in the contrast increased image the algorithm stops. Otherwise, the contrast is gradually increased and the process resumed. This adaptive thresholding method permits to obtain robust image edges, as depicted in the examples provided in Figure 5.

**Figure 6.**
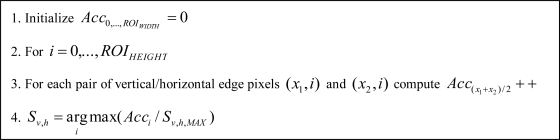
Vertical and/or horizontal edges symmetries computation procedure.

**Figure 7.**
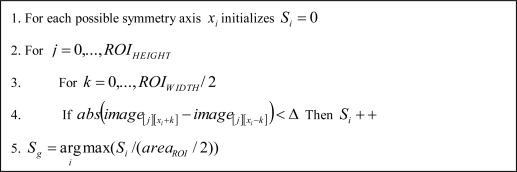
Gray level symmetry computation procedure.

**Figure 8.**
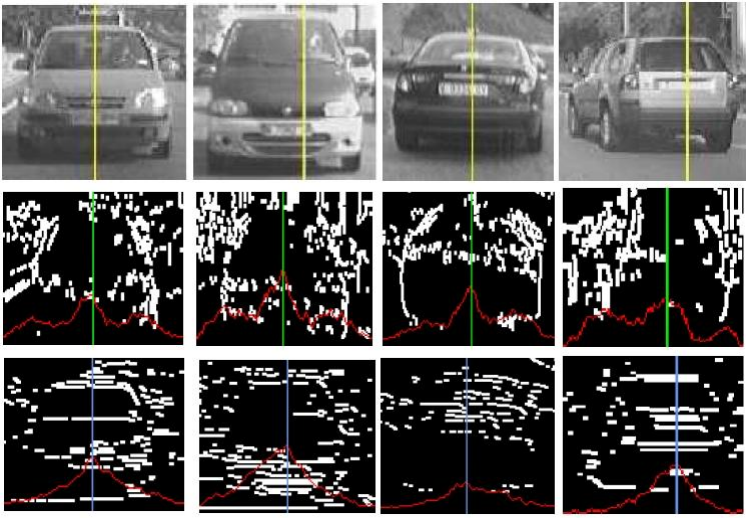
Upper row: gray level symmetry; Middle row: vertical edges symmetry; Lower row: horizontal edges symmetry.

In a second step, vertical edges (*S_v_*), horizontal edges (*S_h_*) and grey level (*S_g_*) symmetries are obtained, so that, candidates will only pass to the next stage if their symmetries values are greater than a threshold. The vertical and horizontal edges symmetries are computed as listed in Figure 6. The grey level symmetry computation procedure is shown in Figure 7. Some examples of the three types of symmetries are depicted in Figure 8.

**Figure 9.**
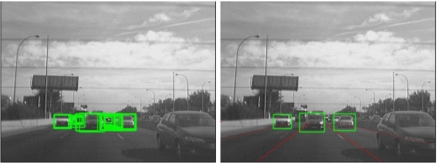
Left: overlapped candidates. Right: non-maximum suppression results.

Symmetry axes are linearly combined to obtain the final position of the candidate. Finally, a weighted variable is defined as a function of the entropy of Canny points, the three symmetry values and the distance to the host vehicle. We use this variable to apply a non-maximum suppression process per lane which removes overlapped candidates. An example of this process is shown in Figure 9.

**Figure 10.**
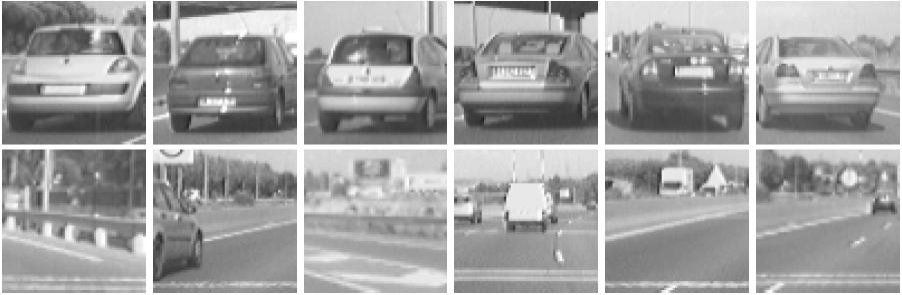
Forward data set. Upper row: positive samples (vehicles); Lower row: negative samples.

**Figure 11.**
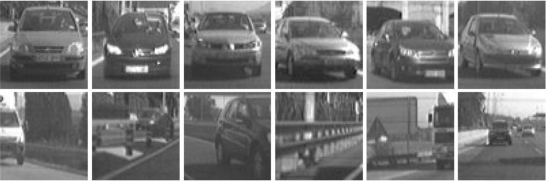
Rear data set. Upper row: positive samples (vehicles); Lower row: negative samples.

**Figure 12.**
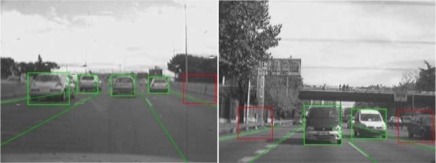
Linear SVM with HOG features single-frame classification examples: non-vehicles are depicted with red boxes whereas vehicles are depicted with green ones.

The selected candidates are classified by means of a linear Support Vector Machine (SVM) classifier [13], in combination with Histograms of Oriented Gradients features [14]. We have developed and tested two different classifiers depending on the module (forward and rear classifiers). All candidates are resized to a fixed size of 64 × 64 pixels to facilitate the features extraction process. The rear-SVM classifier is trained with 2,000 samples and tested with 1,000 samples (1/1 positive/negative ratio) whereas the forward-SVM classifier is trained with 3,000 samples and tested with 2,000 samples (1/1 positive/negative ratio). Figures 10 and 11 depict some positive and negative samples of the forward and rear training and test data sets respectively. Figure 12 shows a couple of examples of vehicle detection after linear SVM classification with HOG features.

**Figure 13.**
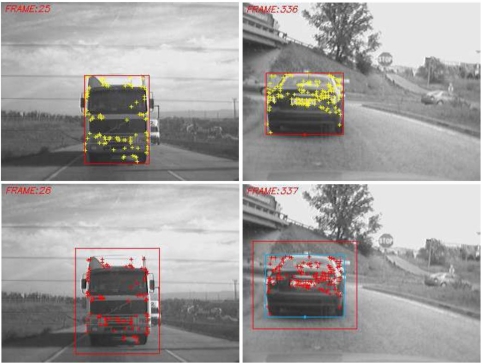
Data association by features matching. Upper row: Harris features on image *t*. Lower row: matched Harris features on image *t* + 1.

After detecting consecutively an object classified as vehicle a predefined number of times (empirically set to 3 in this work), data association and tracking stages are triggered. The data association problem is addressed by using feature matching techniques. Harris features are detected and matched between two consecutive frames, as depicted in Figure 13.

Tracking is implemented using Kalman filtering techniques [10]. For this purpose, a dynamic state model and a measurement model must be defined. The proposed dynamic state model is simple. Let us consider the state vector *x_n_*, defined as follows:

(4) $$x_{n}={\left[u,\;v,\;w,\;h,\;\dot{u},\;\dot{v},\;\dot{w},\;\dot{h}\right]}^{T}$$

In the state vector *x* and *y* are the respective horizontal and vertical image coordinates for the top left corner of every object, and *w* and *h* are the respective width and height in the image plane. A dynamical model equation can be written like this:

(5) $$x_{n+1}=A\cdot x_{n}+\omega_{n}=\left(\begin{array}{cccccccc} 1 & 0 & 0 & 0 & \Delta t & 0 & 0 & 0\\ 0 & 1 & 0 & 0 & 0 & \Delta t & 0 & 0\\ 0 & 0 & 1 & 0 & 0 & 0 & \Delta t & 0\\ 0 & 0 & 0 & 1 & 0 & 0 & 0 & \Delta t\\ 0 & 0 & 0 & 0 & 1 & 0 & 0 & 0\\ 0 & 0 & 0 & 0 & 0 & 1 & 0 & 0\\ 0 & 0 & 0 & 0 & 0 & 0 & 1 & 0\\ 0 & 0 & 0 & 0 & 0 & 0 & 0 & 1 \end{array}\right)\;{\left(\begin{array}{c} u\\ v\\ w\\ h\\ \dot{u}\\ \dot{v}\\ \dot{w}\\ \dot{h} \end{array}\right)}_{n}+\omega_{n}$$

In the model, Δ*t* is the simple time, *A* represents the system dynamics matrix and *ω_n_* is the noise associated to the model. Although the definition of A is simple, it proves to be highly effective in practice since the real time operation of the system permits to assure that there will not be great differences in distance for the same vehicle between consecutive frames. The model noise has been modelled as a function of distance and camera resolution. The state model equation is used for prediction in the first step of the Kalman filter. The next step is to define the measurement model. The measurement vector is defined as *z_n_* = [*u, v, w, h*]*^T^*. Then, the measurement model equation is established as follows:

(6) $$z_{n+1}=H\cdot x_{n}+v_{n}=\left(\begin{array}{cccccccc} 1 & 0 & 0 & 0 & 0 & 0 & 0 & 0\\ 0 & 1 & 0 & 0 & 0 & 0 & 0 & 0\\ 0 & 0 & 1 & 0 & 0 & 0 & 0 & 0\\ 0 & 0 & 0 & 1 & 0 & 0 & 0 & 0 \end{array}\right)\;{\left(\begin{array}{c} u\\ v\\ w\\ h\\ \dot{u}\\ \dot{v}\\ \dot{w}\\ \dot{h} \end{array}\right)}_{n}+v_{n}$$

In last equation H represents the measurement matrix and *v_n_* is the noise associated to the measurement process. The purpose of the Kalman filtering is to obtain a more stable position of the detected vehicles. Besides, oscillations in vehicles position due to the unevenness of the road makes *v* coordinate of the detected vehicles change several pixels up or down. This effect makes the distance detection unstable, so a Kalman filter is necessary for minimizing these kinds of oscillations.

### 2.4. Error analysis

Accurate detection of the wheel-to-road contact point of the preceding vehicle is essential for assuring maximum precision of the host-to-vehicle estimated distance. Thus, the error committed in estimating the host-to-vehicle distance *Z_err_* due to a vehicle detection error of *n* pixels in the image plane is given by:

(7) $$Z_{\mathrm{err}}=Z_{n}-Z=\frac{f_{v}\;h_{\mathrm{CAM}}}{v+n}-Z=\frac{-{\mathrm{nZ}}^{2}}{f_{v}\;h_{\mathrm{CAM}}+\mathrm{nZ}}$$

where *v* is the vertical coordinate of the wheel-to-road contact point in the image plane, *Z* is the estimated host-to-vehicle distance, *f_v_* is the vertical focal length in pixels and *h_CAM_* represents the elevation of the camera above the ground. Considering an error of one pixel *n* = 1 and *f_v_h_CAM_* ≫ *nZ*, *Z_err_* becomes:

(8) $$Z_{\mathrm{err}}\approx \frac{{\mathrm{nZ}}^{2}}{f_{v}\;h_{\mathrm{CAM}}}$$

For example, for a 320 × 240 image, a focal length *f_v_* = 370 *px*, and a camera height *h_CAM_* = 1.2*m*, an error of 1 pixel (*n* = 1) becomes a relative 5% error at a distance:

(9) $$Z=\frac{Z_{\mathrm{err}}}{Z}\;f_{v}\;h_{\mathrm{CAM}}=0.05\times 370\times 1.2=22.2m$$

**Figure 14.**
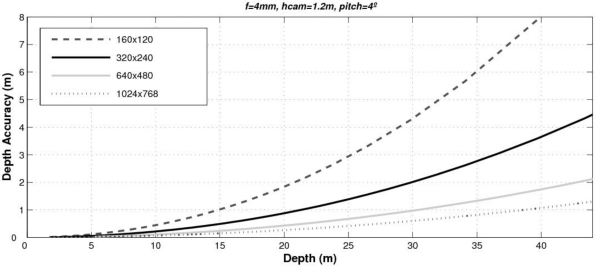
Accuracy of the host-to-vehicle distance obtained by using different images resolution.

On the other hand, the error at 44.4 m is 10%. In Figure 14 we can see the depth accuracy due to quantization for different images resolutions. As can be seen, the larger the images resolution the better the accuracy. Unfortunately, a trade off must be reached between the accuracy of the depth measurements and the computational costs. In our case the size of the images is 320 × 240 pixels which provides accuracy more than enough for automotive applications.

The distance measurements are used to obtain the relative host-to-vehicle velocity. Relative velocity *v_H2V_* is computed using the following equation:

(10) $$v_{H2V}=\frac{\Delta Z}{\Delta t}$$

Based on the scale change *s* of detected objects in the image plane, the optimal value of Δ*t* that minimizes the estimation noise can be calculated. Let *W* denote the width (in meters) of the preceding vehicle, *w* and *w′* the width of the preceding vehicle in the image plane when it is located at distances *Z* and *Z′*, respectively, with regard to the host vehicle. The scale change *s* can be defined as:

(11) $$s=\frac{\omega -\omega \prime }{\omega \prime }$$

Then, the estimated relative velocity can be computed as follows:

(12) $$v_{H2V}=\frac{\Delta Z}{\Delta t}=\frac{Z\frac{\omega -\omega \prime }{\omega \prime }}{\Delta t}=\frac{\mathrm{Zs}}{\Delta t}$$

As demonstrated in [15], the value of Δ*t* that minimizes the error in the estimated relative velocity is given by:

(13) $$\Delta t=\sqrt{\frac{2Z^{2}s_{\mathrm{err}}}{f_{v}\;\mathrm{Wa}}}$$

where *a* represents the acceleration of the host vehicle, and *s_err_* is the error committed in the estimation of scale change. Building on this result, the optimal value of Δ*t* for zero acceleration is infinite. In practice, it has been limited to Δ*t* = 1.0 s, which matches with both the GPS and the CAN bus sample time (1 Hz).

### 2.5. Traffic load and road speed

As depicted in Figure 2, the Traffic Data Collection module uses three sources of data: the measurements provided by the GPS, the data supplied by the CAN bus (vehicle speed) and the outputs obtained from both vision-based vehicle detection systems. Whereas the GPS and the CAN bus sample frequency is 1 Hz, the vision-based system operates in real-time at 25 frames per second (25 Hz). In order to obtain measurements from GPS and CAN bus at 25 Hz we apply a linear interpolation between two consecutive samples.

The outputs of the forward and rear vehicle detection systems at frame *i* are the number of detected vehicles *N_i_* and their corresponding distances to the host vehicle $d_{i}^{\left(k\right)}$. These outputs are combined to cover the local environment of the vehicle. The traffic load at frame *i* is given by next equation:

(14) $$L_{i}=(N_{i}+1)/N_{\mathrm{MAX}}$$

where *N_MAX_* is the maximum number of vehicles in range that can be detected by both systems (in our case *N_MAX_* is defined as 8 or 12 for two lanes and three lanes roads respectively). The average road speed at frame *i* is computed as follows:

(15) $$v_{i}=\frac{1}{(N_{i}+1)\Delta t}\;\sum_{k=0}^{N_{i}-1}\;\left(d_{i}^{\left(k\right)}-d_{i-1}^{\left(k\right)}\right)+v_{i}^{h}$$

where $d_{i}^{\left(k\right)}$ and $d_{i-1}^{\left(k\right)}$ represent the distance between the host vehicle and vehicle *k* at frames *i* and *i* − 1 respectively, Δ*t* corresponds to the sample time (which is limited to 1.0 s as described in Section 2.4), $v_{i}^{h}$ is the host vehicle speed provided by the CAN bus, and *N_i_* is the number of detected vehicles. Note that the distance values correspond to filtered measurements since they are obtained from the first two elements of the Kalman filter state vector (*u* and *v*) using known camera geometry and ground-plane constraints.

## 3. Experiments

The system was implemented on a PC Core 2 Duo at 3.0 GHz and tested in real daytime traffic conditions using CMOS cameras in the visible spectrum with low resolution images (320 × 240). After training and test, a trade-off point has been chosen at Detection Rate (DR) of 95% and False Positive Rate (FPR) of 5% for the rear-SVM classifier and at DR of 90% and FPR of 6% for the forward-SVM classifier. We have to note that these numbers are obtained in an off-line single-frame fashion, so that, they will be improved in subsequently stages. In addition, the lane detection system reduces the searching area and the number of false candidates passed to further stages.

**Figure 15.**
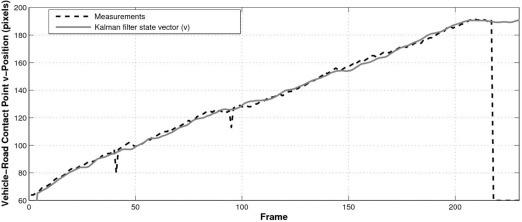
Estimated wheel-to-road contact point and the corresponding Kalman filter output.

The benefits of using the proposed Kalman filter model can be seen in Figure 15, which plots the measured wheel-to-road contact point and the corresponding filtered value. As can be observed the use of a Kalman filter absorbs spurious detection problems and allows tracking the vehicle for a few frames once it has been lost by the detection stage.

**Figure 16.**
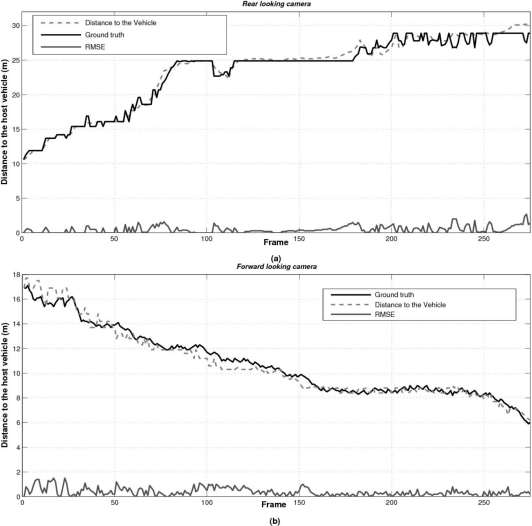
Estimated host-to-vehicle distance by the proposed sensor, ground truth and absolute error in two examples corresponding to (a) rear and (b) forward vision modules.

**Figure 17.**
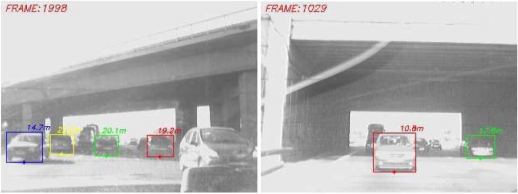
Examples with strong illumination changes after passing beneath a bridge.

In order to evaluate the accuracy of the host-to-vehicle distances estimated by the proposed approach we have generated a ground truth by manually labelling the position of the vehicles in the images, in a frame by frame process. Thus we can compute the root mean square error (RMSE). The obtained results for the forward and rear modules can be observed in Figures 16a,b, respectively. Due to perspective constraints and the discrete nature of the sensor, the larger the host-to-vehicle distance the larger the error. The largest errors take place in cases where the host vehicle is passing beneath a bridge due to strong illumination changes (see Figure 17). The overall RMSE is 0.47 m for the forward example and 0.39 m for the rear one, which are acceptable for automotive applications.

**Figure 18.**
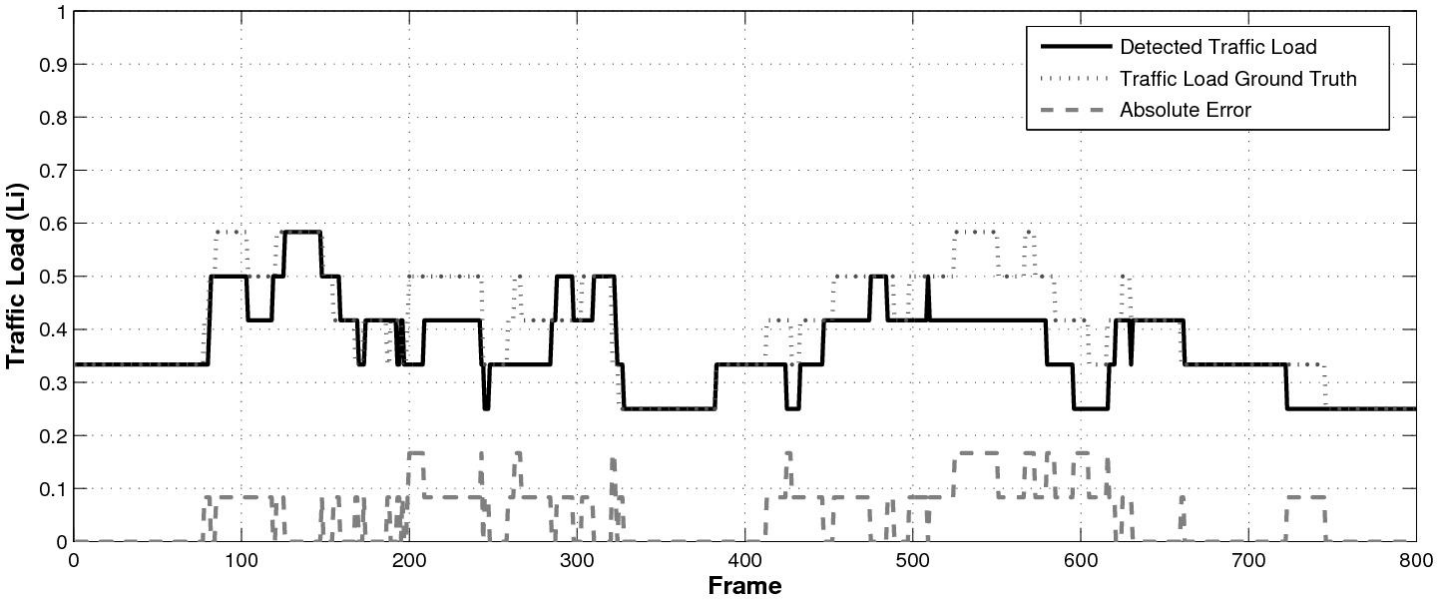
Estimated traffic load (*L_i_*) using Equation 14 and the corresponding ground truth.

Finally, in order to validate the proposed sensor for traffic collection in automotive applications we have recorded several video sequences in real traffic conditions and we have manually labeled the number of vehicles in range at every frame (a total of 800 frames). The speed of the host vehicle was around 90 km/h so the length of the traveled route was approximately 1 km. Both the traffic load *L_i_* and the average road speed *v_i_* are computed at every frame using Equations 14 and 15. Figure 18 shows the estimated traffic load, the ground truth and the corresponding absolute error. The overall RMSE in the traffic load computed by the proposed approach is 0.07 (7%).

**Figure 19.**
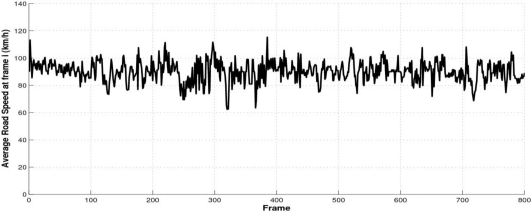
Estimated average road speed (*v_i_*) using Equation 15.

The average road speed *v_i_* at every frame is depicted in Figure 19. Most of the errors occur in images with strong illumination changes, in curves and in cases where there are strong changes in the vehicle pitch, roll as well as the camera height.

## 4. Conclusions

This paper presented a traffic data collection system for the automotive industry which comprises one rear and one forward looking cameras, covering a nearly 360 degree field of view. The proposed sensor provides accurate host-to-vehicle distance measurements in daytime conditions in a four stage process (*lane detection*, *candidates selection*, *vehicles classification* and *tracking*), with an average error lower than 0.5 m, which is more than enough for automotive applications such as platooning, ACC, collision avoidance/mitigation, traffic monitoring, *etc*., without the need of other sensors [8]. Due to both perspective constraints and the discrete nature of the sensor, the larger the host-to-vehicle distance the larger the error. However, the accuracy of the measurements increases in proportion to the collision risks, i.e., as long as the host-to-vehicle distances decrease. The sensor also computes measurements concerning relative host-to-vehicle velocities, traffic load and average road speed, by combining the outputs of the vision modules with the data supplied by the CAN bus and the GPS sensor. The overall error of the computed traffic load is around 7%. Compare to previous extended FCD systems [7] the proposed approach provides a more representative description of the local traffic conditions of the host vehicle, since it covers a nearly 360 degree field of view.

Most of the errors are due to strong illumination changes and variations in the extrinsic relationship between the camera and the road (pitch, roll and camera height). As future work, we are planning to reduce these errors by including accurate estimation of the ego-motion of the vehicle relative to the road using input from both the CAN bus and the cameras. In addition, new experiments will be planned to perform traffic data collection in night time conditions by including active illumination or infrared cameras.
